# Danggui Shaoyao San Ameliorates Renal Fibrosis via Regulation of Hypoxia and Autophagy

**DOI:** 10.1155/2019/2985270

**Published:** 2019-03-17

**Authors:** Meng-yun Zhang, Hui-hua Chen, Jing Tian, Hui-juan Chen, Ling-ling Zhu, Pei Zhao, Ting Zhang

**Affiliations:** ^1^Teaching and Research Department of Basic Theory of Traditional Chinese Medicine, Shanghai University of Traditional Chinese Medicine, 1200 Cailun Road, Shanghai 201203, China; ^2^The Public Experiment Platform, Shanghai University of Traditional Chinese Medicine, 1200 Cailun Road, Shanghai 201203, China; ^3^Department of Pathology, Shanghai University of Traditional Chinese Medicine, 1200 Cailun Road, Shanghai 201203, China

## Abstract

Danggui Shaoyao San (DSS), a traditional Chinese medicinal prescription, was widely used to reinforce earth to activate collaterals in ancient times. Recently, many clinical studies found that DSS had a renoprotection. In this study, we evaluated the effect of DSS on unilateral ureteral obstruction- (UUO-) induced renal fibrosis in rats and investigated the mechanisms underlying the effect. Sprague Dawley (SD) rats were randomized to UUO or Sham operation. After 1 day, the rats that underwent UUO were randomized to treatment for four experimental groups (n=10 each group): Sham, UUO only, UUO+ benazepril (Bena), and UUO+DSS. After 4 weeks, we demonstrated that DSS significantly suppressed UUO-induced renal hypertrophy by gravimetric. In addition, DSS obviously prevented UUO-induced disorder in renal structure and renal function by HE and biochemistry test. We also found that DSS abrogated UUO-induced renal fibrosis by Masson's staining and collagen volume fraction (CVF) analysis; this is consistent with the western blot analysis that showed DSS abrogated the UUO-induced enhanced TGF-*β*1 and weakened BMP-7. Compared with the UUO only group, rats treated with DSS exhibited significant increase in vascular density, followed by decrease in hypoxia and HIF-1*α* protein level through western blot and immunofluorescence analysis. Furthermore, we also determined proteins of autophagy and DSS enhanced autophagy to prevent the damage-induced by UUO. Taken together, our findings demonstrated that DSS had a renoprotection effect in ameliorating renal fibrosis possibly via attenuating tissue hypoxia and regulating autophagy.

## 1. Introduction

Chronic kidney disease (CKD) is characterized by abnormalities of kidney structure or function, and the prevalence of CKD is rising greatly in recent years and it has become a worldwide heath issue [1]. The eventual outcome of CKD may be end-stage kidney disease (ESKD), when patients need dialysis or transplantation to survive. Diabetes, hypertension, and primary glomerulonephritis are the most common causes of CKD. However, the final common pathology of CKD is renal interstitial fibrosis (RIF). Fibrosis is a pathological state characterized by excess extracellular matrix (ECM) protein production and progressive accumulation. Transforming growth factor-*β* (TGF-*β*) has long been well known as potent regulator of ECM production and fibrogenesis, and they are induced during kidney injury. In renal biopsy samples from patients with CKD, some evidences typically suggested loss of peritubular capillaries in areas of tubulointerstitial fibrosis, leading to a reduction in oxygen supply. Chronic hypoxia has been recognized to play a pivotal role in CKD [2]. Hypoxia is a profibrogenic stimulus for tubular cells, interstitial fibroblasts and renal microvascular endothelial cells. Tubular cells under hypoxic conditions undergo epithelial-mesenchymal transdifferentiation, leading to fibrosis [3]. Hypoxia has been proposed as an important microenvironmental factor in the development of tissue fibrosis. Hypoxia-inducible factor (HIF) is an important regulatory factor that allows individual cells to adapt to hypoxia, which play an important role in normal tissue repair processes, but in some cases of chronic injury, chronic hypoxia, and pathological repair, the hypoxia pathway might be responsible for driving the process of fibrosis and could lead to excessive scarring and compromised organ function [3]. In CKD, fibrosis presumably also further aggravated hypoxia by impairing oxygen diffusion and supply to tubular and interstitial cells through increasing the distance between the capillaries and tubules [4]. Meanwhile, hypoxia subsequently triggered tubular injury and interstitial fibrosis; these in turn damaged adjacent capillaries, which exacerbated fibrosis of the kidney and subsequent chronic hypoxia, and set in motion a vicious cycle. Autophagy is a cellular process of degradation of damaged cytoplasmic components. Dysregulated autophagy had been implicated in disorders characterized by fibrosis in various tissues, including liver fibrosis, cardiac fibrosis, and renal fibrosis [5]. Autophagy had been reported to regulate renal fibrosis but its role on renal fibrosis remains unclear. In UUO model, autophagy has a protective role in renal fibrosis [6]. Some researches [7–9] demonstrated that autophagy can regulate TGF-*β* expression and suppress kidney fibrosis in UUO model using LC3^−/−^ mice and Beclin 1 heterozygous mice. In contrast, another study [10] explained that the persistent activation of autophagy promotes renal tubulointerstitial fibrosis during UUO. As we all known, UUO produces ischemic and hypoxic stress to tubular cells, autophagy can be induced by hypoxia; in our study, we want to investigate the regulation of autophagy.

Danggui Shaoyao San (DSS), which consists of Danggui, Baishao, Chuanxiong, Fuling, Baizhu, and Zexie, was originally recorded in “Jingui Yaolue”, which was written by Zhongjing Zhang in the Eastern Han Dynasty. It has a long history of treating gynecological disorders since ancient times in China. In recent years, Liu's study [11] showed that DSS partially decreased the high plasma glucose level and attenuated the increased expression of NF-*κ*B as well as TGF-*β*1 and the progressive accumulation of type IV collagen; these data suggested DSS had the renoprotective effects in STZ-diabetic rats. In the clinic, we also found that DSS had a renoprotection, but the pharmacological mechanism of its action is still under investigation. The aim of this study was to document the beneficial effect of DSS on UUO-induced renal fibrosis in the rats and to investigate the mechanism of action.

## 2. Materials and Methods

### 2.1. Preparation of Danggui Shaoyao San (DSS)

DSS is composed of Danggui, Baishao, Chuanxiong, Fuling, Baizhu, and Zexie in a dry weight ratio of 10:30:9:20:20:15. All herbs were supplied by the Shanghai Kangqiao Chinese Medicine Tablet Co., Ltd. (Shanghai, China) (Table 1). The decoction pieces mixture was added with water of 4 times volume, boiled 2 times, concentrated using a rotary evaporator, and obtained the equivalent crude content of 2.35g/ml. The dosage of DSS represents the dry weight of the raw herbs used to produce decoction. 6.3 times of the normal dosage for adult human was defined as the dosage of DSS for rats. Thus, rats in DSS groups were given 9.4g/kg/day of DSS.

### 2.2. Experimental Animals

The study protocol was approved by the Animal Care and Use Committee of Shanghai University of Traditional Chinese Medicine. Forty SD male rats weighing 200±20g were housed in cages with controlled temperature and humidity, 12 h light-dark periods, and free access to water and a standard diet. Prior to the experiments, all rats were given a period of about 7 days for acclimatization.

### 2.3. UUO Surgery

UUO surgery was used to generate a rat model of progressive chronic renal fibrosis [12]. Briefly, the rats were placed in a chamber, anesthetized with 5% isoflurane (Surgivet, MI, USA). The rats were placed supine on a heating pad. Skin preparation and disinfection were performed. The obstructed side of ureter was visualized following a flank incision and ligated twice with 4.0 silk suture, one near the lower pore of the kidney and another below the first ligation. The ureter was cut between two ligations. The incision was closed by suture layer. The animals in Sham group underwent all operation procedures except the ligation and cut.

### 2.4. Treatments

After the UUO surgery or Sham operation for 1 day, the rats were randomly divided into four experimental groups (n=10 for each group): Sham, UUO only, UUO+benazepril (Bena, 1.7 mg/kg), UUO+DSS (9.4g/kg). The Bena, DSS, or normal saline (for the Sham operation and UUO only groups) was provided once a day by oral gavage for 4 weeks.

### 2.5. Assays for Renal Functions

Blood samples were collected from the abdominal aortic under anesthesia. The serum was separated by centrifugation at 3000×*g* (4°C) for 10 min, and blood urea nitrogen (BUN), creatinine, and UA were detected using an automatic biochemical analysis system (Hitachi, Ltd., Tokyo, Japan).

### 2.6. Collection of Tissue Sample

At last, the rats were all sacrificed and kidneys on the obstructed side were harvested, rinsed briefly in phosphate-buffered saline (PBS), and blotted dry, and the body weight, kidney weight, and tibia length were measured; then the kidney specimen was cut open sagittally, half of which was fixed for morphological and immunofluorescence, and the remaining half was stored at −80°C for the detection of related proteins.

### 2.7. H&E Staining and Masson's Trichrome Staining

Tissue samples were fixed in 4% paraformaldehyde overnight at 4°C, rinsed, and transferred to PBS, followed by paraffin embedding, and then serially sectioned at a thickness of 5 *μ*m for histologic analysis. Sections were stained with hematoxylin and eosin, following standard procedures; then the sections were investigated using an optical microscope (Stemi DV4 or Axio Scope A1, Carl Zeiss, Oberkochen, Germany). Fibrosis was evaluated by Masson's trichrome staining and collagenous fibers stained blue and the other tissue stained red. Collagen deposition was quantitatively analyzed as collagen volume fraction (collagen area/total area × 100%), which was determined by using Image-Pro Plus 6.0 (Media Cybernetics, Inc., Silver Spring, MD, USA).

### 2.8. Immunofluorescence Staining

For immunofluorescence assay, the paraffin-embedded renal section (5*μ*m) was deparaffinized and rehydrated and then suffered antigen retrieval. Nonspecific protein was blocked with 5% fetal bovine serum for 60 min at room temperature (RT). Then sections were incubated with Tomato Lectin (Lycopersicon Esculentum Lectin, LEL, Vector Laboratories, Inc., Burlingame, CA, USA), FITC-MAb1 antibody (HP2 Hypoxyprobe™-1 Plus Kit, Hypoxyprobe, Inc., Burlington, MA, USA) at RT for 1 h, respectively, and images were obtained at 465 nm excitation by a Carl Zeiss LSM800 confocal microscope (Carl Zeiss Microscope GmbH, Jena, Germany). Sections were also incubated with a primary antibody against HIF-1*α* (Abcam, Cambridge, CB, UK) and then stained with DAPI (Sigma, St.Louis, MO, USA), followed by Alexa Fluor 555 nm (red, CST, Danvers, MA, USA).

### 2.9. Western Blotting Analysis

Kidney tissue protein lysates were separated by 10% SDS-PAGE and then transferred to PVDF (0.45*μ*m, EMD Millipore, Billerica, MA, USA). The membranes were probed overnight at 4°C with primary antibodies against HIF-1*α* (Abcam, Cambridge, CB, UK), BMP-7 (Abcam, Cambridge, CB, UK), TGF-*β*1 (Santa Cruz Biotechnology, Dallas, TX, USA), LC3II/I (CST, Danvers, MA, USA), p62 (CST, Danvers, MA, USA), and GAPDH (Proteintech, Rosemont, IL, USA). The next day, the membranes were washed and incubated with the appropriate secondary antibody for 1 h at room temperature. After the final wash, signals were detected using the FluorChem E imaging system (ProteinSimple, San Francisco, CA, USA) and a chemiluminescence detection kit (EMD Millipore). Protein band densities were quantified by using an image analysis system (Alpha View SA, ProteinSimple, San Francisco, CA, USA) and expressed as ratios to GAPDH.

### 2.10. Statistical Analysis

Data were analyzed using SPSS21.0, and results were presented as means ±SEM. Data from the experimental groups were compared by one-way analysis of variance (ANOVA) followed by Tukey's post hoc analysis, and differences were considered significant at* P*<0.05.

## 3. Resuls

### 3.1. DSS Suppressed UUO-Induced Hypertrophy in Kidney

**Figure 1:**
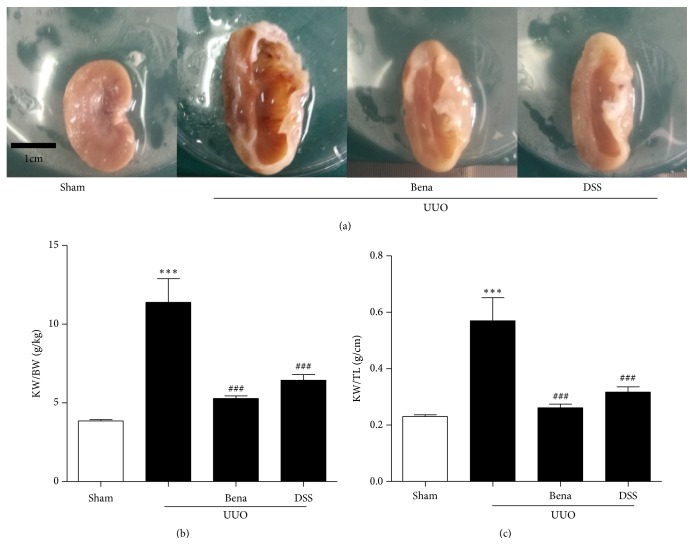
*DSS suppressed UUO-induced increasing in kidney weight*. (a) Comparison of kidney size (scale bar, 1 cm) in rats that underwent the Sham, UUO only, or UUO followed by treatment with Bena or DSS. (b) and (c) Ratios of kidney eight-to-body weight and kidney weight-to-tibial length in the four treatment groups. Results are expressed as mean SEM. ^*∗∗∗*^*P* < 0.001 versus Sham; ^###^*P*<0.001 versus UUO only. UUO, unilateral ureteral obstruction; Bena, Benazepril, 1.7 mg/kg/day by oral gavage for 4 weeks; DSS, Danggui Shaoyao San, 9.4g/kg/day by oral gavage for 4 weeks; KW, kidney weight; BW, body weight; TL, tibial length.

To determine the effect of DSS on UUO-induced variation of kidney weight, the kidney weight (KW) to body weight (BW) ratio and KW-to-tibial length (TL) ratio were calculated. Compared to the Sham group, rats in the UUO only group exhibited considerable hypertrophy of the kidney (Figure 1(a)) and higher KW/BW and KW/TL ratio (Figures 1(b) and 1(c)). In contrast, the kidney in DSS-treated group was considerably smaller and significantly reversed the UUO-induced increase in the KW/BW and KW/TL ratio.

### 3.2. DSS Improved UUO-Induced Renal Dysfunction

**Figure 2:**
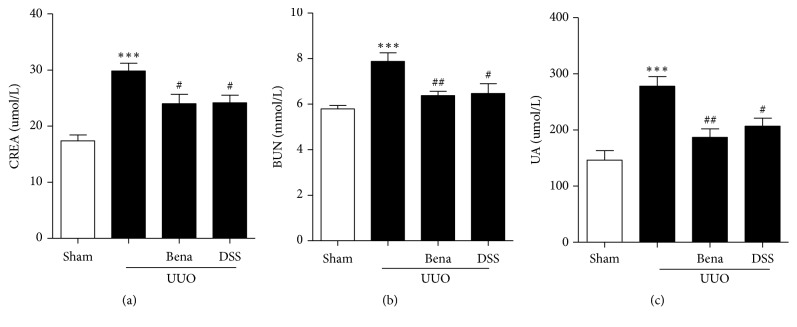
*Effect of DSS on renal functions induced by UUO*. (a) Serum creatinine. (b) Serum urea nitrogen. (c) Serum uric acid. Results are expressed as mean SEM. ^*∗∗∗*^*P* < 0.001 versus Sham; ^#^*P*<0.05, ^##^*P*<0.01, and ^###^*P*<0.001 versus UUO only. UUO, unilateral ureteral obstruction; Bena, Benazepril, 1.7 mg/kg/day by oral gavage for 4 weeks; DSS, Danggui Shaoyao San, 9.4g/kg/day by oral gavage for 4 weeks; CREA, creatinine; BUN, serum urea nitrogen; UA, uric acid.

To assess renal function, serum creatinine (CREA), blood urea nitrogen (BUN), and serum uric acid (UA) tests were determined. The results showed that, in the UUO only group, CREA, BUN, and UA values were higher than those in the Sham group and the difference was statistically significant. However, the levels of those indexes were significantly decreased in rats that received DSS after UUO (Figures 2(a), 2(b), and 2(c)). These results suggested that DSS treatment protected against UUO-induced renal dysfunction.

### 3.3. DSS Improved UUO-Induced Renal Morphological Changes

**Figure 3:**
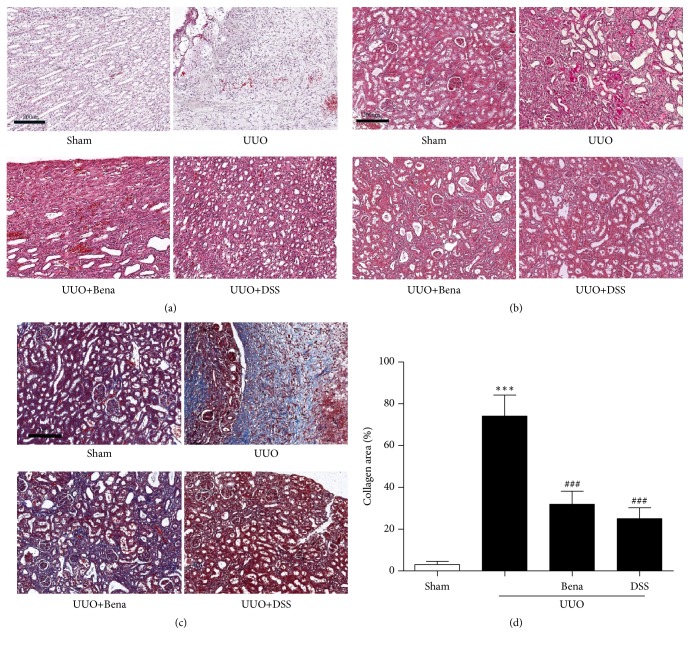
*Effect of DSS on UUO-induced kidney pathological changes and fibrosis in rats*. ((a) and (b)) H&E showing pathological changes in medulla and cortex. (c) Masson's trichrome staining. (d) Quantification of collagen volume fraction (collagen area/total area × 100%). Results are expressed as mean ± SEM. ^*∗∗∗*^*P* < 0.001 versus Sham; ^###^*P*<0.001 versus UUO only. UUO, unilateral ureteral obstruction; Bena, Benazepril, 1.7 mg/kg/day by oral gavage for 4 weeks; DSS, Danggui Shaoyao San, 9.4g/kg/day by oral gavage for 4 weeks.

As shown in the Figure 3, H&E staining was used to evaluate the pathological changes of renal tissue. Compared with the Sham group, we discovered that in the UUO only group, the structure of collecting tubule was disorder and fibrosis seriously in the medulla and the cortex of the obstructed side showed significant tubular dilation, tubular atrophy, and atrophic tubules were filled with proteinaceous casts, accompanied with inflammatory cell infiltration and fibrosis in the renal interstitium (Figures 3(a) and 3(b)). However, rats treated with DSS after UUO showed considerably normal in collecting tubule and glomerular, less inflammatory cell infiltration, and obvious fibrosis was not observed in the renal interstitium. To evaluate fibrosis in the kidney, Masson's trichrome staining was used to stain for collagen or fibrosis where blue represents the collagen deposition (Figures 3(c) and 3(d)). Compared with the Sham group, the UUO only group showed widespread fibrous tissue, which is consistent with the western blot analysis (Figure 5(a)) that showed enhanced TGF-*β*1 and weakened BMP-7. However, rats treated with DSS after UUO showed considerably less fibrous tissue. Obviously, DSS could prevent the rise in TGF-*β*1 and decline in BMP-7 induced by UUO, suggesting that DSS is able to attenuate renal fibrosis.

### 3.4. Upregulation of Vascular Density and Improvement of Tissue Hypoxia Probably Contributes to the Protective Effect of DSS

**Figure 4:**
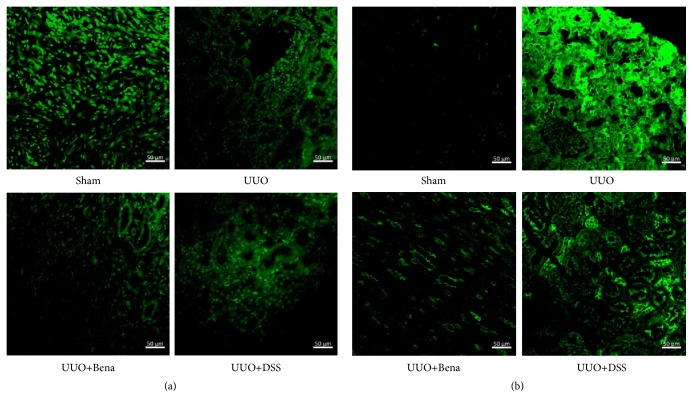
*Effect of DSS on vascular density and hypoxia*. (a) Representative confocal fluorescent images of the kidney sample with Tomato Lectin (green). (b) Representative confocal fluorescent images of the kidney sample with Hypoxyprobe (green). UUO, unilateral ureteral obstruction; Bena, benazepril, 1.7 mg/kg/day by oral gavage for 4 weeks; DSS, Danggui Shaoyao San, 9.4g/kg/day by oral gavage for 4 weeks.

**Figure 5:**
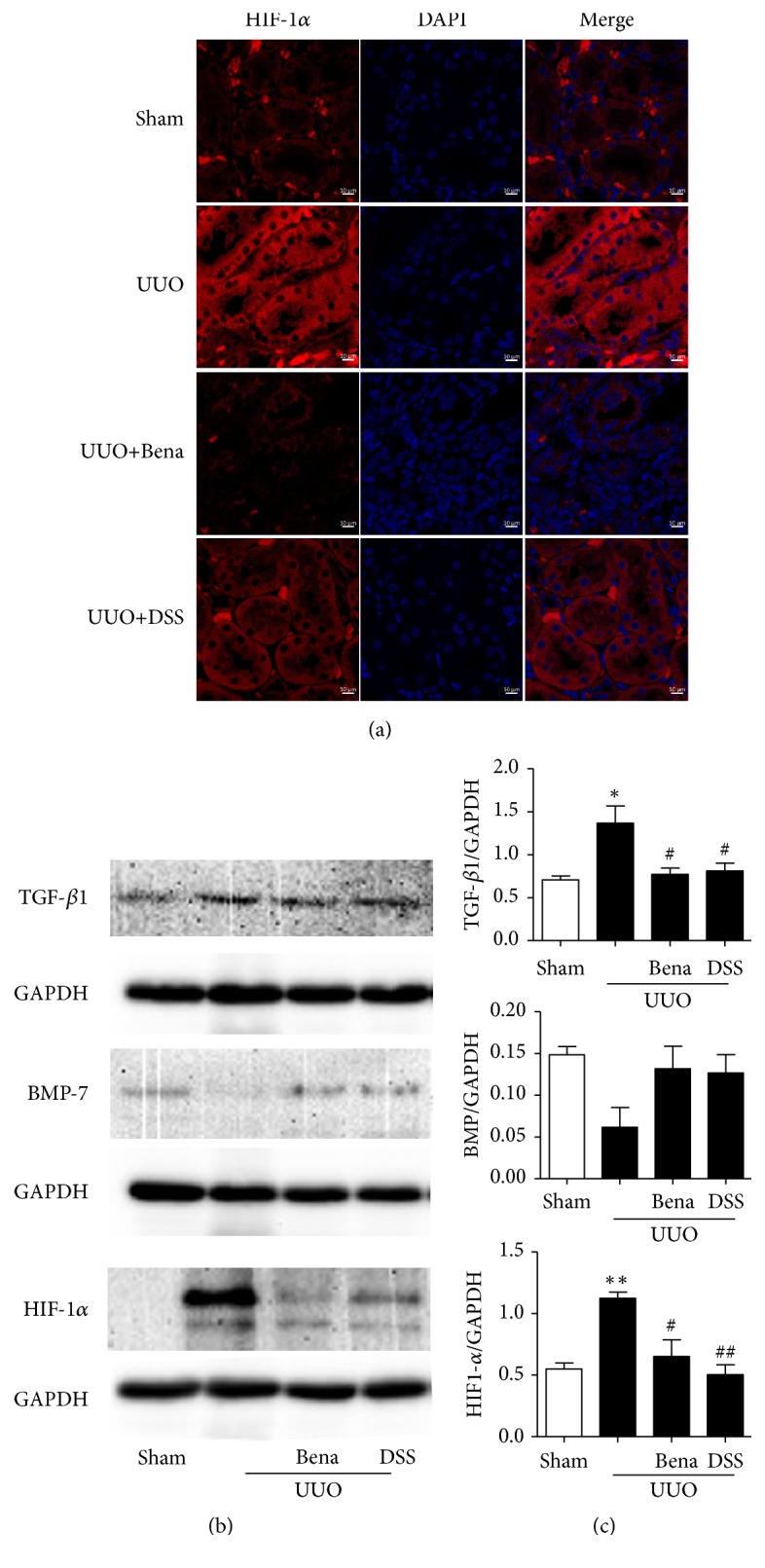
*Effect of DSS on renal fibrosis associated with hypoxia*. (a) Representative confocal fluorescent images of the kidney sample with HIF-1*α* (red) and DAPI (blue). (b) Protein levels of BMP-7, TGF-*β*1, HIF-1*α* in kidney tissue were determined by western blotting (n=3). (c) Quantification of protein levels. Results are expressed as mean SEM. ^*∗∗∗*^*P* < 0.001 versus Sham; ^#^*P*<0.05, ^###^*P*<0.001 versus UUO only. UUO, unilateral ureteral obstruction; Bena, benazepril, 1.7 mg/kg/day by oral gavage for 4 weeks; DSS, Danggui Shaoyao San, 9.4g/kg/day by oral gavage for 4 weeks.

Based on the results described above, we sought to explore the mechanism of renoprotection by DSS. Tomato Lectin is recognized as the most sensitive vessel marker; the fluorescence intensity could reflect the vascular density. We observed that the vascular density decreased significantly in those rats subjected to UUO, which was prevented by DSS (Figure 4(a)). Hypoxyprobe-1 is the hypoxia marker associated monoclonal and polyclonal antibodies that bind to pimonidazole adducts in hypoxia tissue. As shown in Figure 4(b), the fluorescence intensity was increased significantly in rats subjected to UUO only, which suggested serious hypoxia in tissue. HIF-1*α* is recognized as the most important regulator of cellular responses to hypoxia. As shown in Figure 5(a), during hypoxia, the expression of HIF-1*α* increased and translocated into nucleus in the rats subjected to UUO only compared with Sham, which was completely prevented by DSS. These results were consisted with western blot analysis shown in Figure 5(b). Taken together, these observations suggested that DSS prevented the UUO-induced tissue hypoxia.

### 3.5. DSS Probably Enhanced Autophagy to Ameliorate Renal Injury

**Figure 6:**
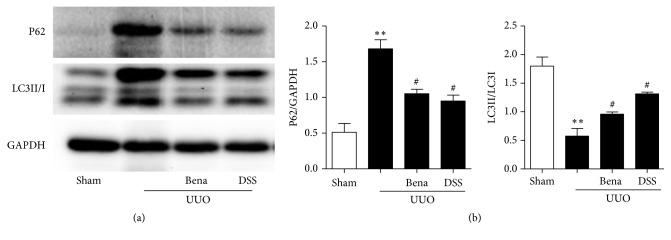
*Effect of DSS on autophagy in rats*. Protein levels of p62 and LC3II/I in kidney tissue were determined by western blotting. (a) Protein bands; (b) quantification of protein levels. Results are expressed as mean SEM. ^*∗∗∗*^*P* < 0.001 versus Sham; ^#^*P*<0.05 and ^###^*P*<0.001 versus UUO only. UUO, unilateral ureteral obstruction; Bena, benazepril, 1.7 mg/kg/day by oral gavage for 4 weeks; DSS, Danggui Shaoyao San, 9.4g/kg/day by oral gavage for 4 weeks.

To determine how DSS regulate autophagy in rats subjected to UUO, we evaluated the LC3II/LC3I ratio by western blot. As shown in Figure 6, the LC3II/LC3I ratio was decreased by UUO, DSS significantly blocked this decrease. Furthermore, we also determined the amount of autophagy cargo p62 that was delivered to lysosomes for degradation. We observed a dramatically increased of p62 in rats subjected to UUO only, which was prevented by DSS. These data indicated that DSS could ameliorate renal injury by increasing autophagy.

## 4. Discussion

The incidence and prevalence of CKD increased greatly in the worldwide, and a considerable proportion of cases eventually progress to end-stage kidney failure, a devastating condition that requires lifelong dialysis or kidney transplantation [13]. Renal fibrosis is regarded as the final common pathway in all forms of CKD and its pathological features include accumulation of ECM as well as extension or atrophy of renal tubule [14]. An approved treatment specifically targeted to renal fibrosis is almost nonexistent [15]. Therefore, there is an urgent need to seek satisfactory therapeutic drugs for renal fibrosis. In clinic, we have found that DSS had a good therapeutic efficacy on CKD. DSS, a famous Chinese formula, has been widely used to treat gynecological disorders since ancient times, but emerging evidences [11, 16–21] have revealed the therapeutic efficacy on various diseases, such as senile dementia, memory loss, depression, Alzheimer's disease, neurodegenerative diseases, and diabetic nephropathy, while the pharmacological mechanism of its action is still unclear. In our study, using the UUO rat model, UUO is a well-established in vivo model of progressive kidney fibrosis. We detected tubular atrophy and interstitial fibrosis, accompanied with an inflammatory cell infiltrate in rats after 4 weeks of UUO. Meanwhile, the function of kidney was worse and the structure subjected to severe damage. But DSS treatment has shown prevention or even reversal of functional and structural changes of the kidney after UUO. During the process of fibrosis, oversecretion of TGF-*β*1 is regarded as a key factor promoting fibrosis [22, 23]. Except that, bone morphogenetic protein-7 (BMP-7) is other protein of the TGF-*β* super family and increasingly regarded as a counteracting molecule against TGF-*β*. A large variety of evidences [24] shows an antifibrotic role of BMP-7 in chronic kidney disease, and this effect is largely mediated via counterbalancing the profibrotic effect of TGF-*β*1. In this study, the UUO group showed histopathological lesions characteristic of kidney fibrosis, as well as high expression of TGF-*β*1 and low expression of BMP-7, while the expression of BMP-7 was increased and TGF-*β*1 was significantly decreased in rats treated with DSS. These data strongly promised a renal protective of DSS in renal fibrosis.

Some evidences [25, 26] displayed loss of peritubular capillaries in areas of tubulointerstitial fibrosis in renal biopsy samples from patients with CKD, leading to a reduction in oxygen supply. Hypoxia is a profibrogenic stimulus for tubular cells, interstitial fibroblasts, and renal microvascular endothelial cells. Tubular cells under hypoxic conditions undergo epithelial-mesenchymal transdifferentiation, leading to fibrosis [3]. Hypoxia has been proposed as an important microenvironmental factor in the development of tissue fibrosis. Thereby, hypoxia has been recognized to play a pivotal role in CKD [27]. HIF is an important regulatory factor that allows individual cells to adapt to hypoxia. In most cases, transient hypoxia and the activation of the HIF-1 pathway are beneficial and promote the repair process. However, in some cases of chronic injury, chronic hypoxia, and pathological repair, the hypoxia pathway might be responsible for driving the process of fibrosis [3, 28–30]. In this study, we tested the vascular density and tissue hypoxia by Tomato Lectin and Hypoxyprobe, respectively. We found that the vascular density obviously decreased in UUO only after 4 weeks and consisted with severe hypoxia, but DSS improved the vascular density and reduced tissue hypoxia. HIF-1 has two faces; although upregulation of HIF-1*α* is protective in acute kidney injury [29], long-term hypoxia-driven overexpression is associated with increased kidney fibrosis. During hypoxia, the expression of HIF-1*α* increased and translocated into nucleus. In our study, the chronic injury was induced after 4 weeks of UUO. We found augment of the expression and translocation of HIF-1*α* in rats after UUO, which could be significantly prevented by DSS.

Autophagy is a cellular process of degradation of damaged cytoplasmic components, which can be induced in response to either intracellular or extracellular factors, such as amino acid or growth factor deprivation, hypoxia, oxidative stress, and organelle damage. LC3 is the best characterized form and the most widely used as an autophagic marker. The conversion of the cytosolic form of LC3 (LC3-I) to lapidated form (LC3-II) indicates autophagosome formation. As we all known, UUO produces ischemic and hypoxic stress to tubular cells and then induces autophagy. But autophagy in the obstructed kidney is a time-dependent manner by UUO; that is, in the early stage of UUO, the level of autophagy increased obviously in the kidney, while, in the late stage of UUO, it decreases significantly [6, 31]. Some studies [7, 10] have shown the activity of autophagy increased while the expression of TGF-*β*1 decreased treated after UUO, which suggested autophagy had a renoprotective role in the obstructed kidney. To investigate the functional role of autophagy in kidney fibrosis, we also examined the two proteins in the kidney after injury induced by UUO. We found the phenomena that after 4 weeks, autophagy was decreased significantly in UUO, and treatment with DSS, the activity of autophagy increased associated with the expression of TGF-*β*1 decreasing. We thought that, in the early stage of UUO, the autophagy activity increased to fight with the injury to protect the kidney, while, in the late stage, the tubular cells may be injured too much to call up the autophagy; the cells may fail in preventing the severe damage from happening.

In recent years, on the basis of traditional Chinese medicine theory, the pathogenesis of renal fibrosis is “deficiency, blood stasis, damp”, and the main therapy were nourishing blood and promoting blood circulation, invigorating spleen, and promoting dampness. With TCM theory, some evidences confirmed that a variety of Chinese herbs and their effective components have the effect of delaying renal fibrosis. According to the theory of TCM, the formula of DSS can be divided into a blood-associated herbs group (Angelica sinensis, Paeonia lactiflora, and Ligusticum chuanxiong) and a water-associated herbs group (Atractylodes macrocephala, Alisma orientale, and Poria cocos), its efficacy is applicable to the treatment principles of renal fibrosis. Zhu [32] found that total glucosides of paeony could retard the process of fibrosis and reduce the pathological damage in adriamycin-induced rats by downregulating the expression of TLR4/NF-*κ*B/TGF-*β*1 signaling. Yuan [33] investigated the effect of ligustrazine (LIG is a purified and chemically identified component of the Chinese herb Ligusticum wallichii Franchat) on renal tubulointerstitial fibrosis using a rat model of unilateral ureteral obstruction, found that LIG significantly reduced the mRNA expression of TGF-*β*1, CTGF, monocyte chemoattractant protein-1, and osteopontin. In vitro, LIG inhibited the TGF-*β*1-induced loss of cytokeratin-18 expression and then could restrain the process of epithelial-myofibroblast transition of tubular epithelial cells. Li 's research [34] found that Zhenwu decoction (including Proria cocos, Atractylodes, and Paeonia lactiflor) can reduce Hyp content in kidney tissue, alleviate histological changes, and improve renal function in adriamycin nephropathy (AN) rats, and it might protect kidney against AN by increasing the expression of podocin and nephrin. In this study, we investigated the compound of DSS; in the future, we would split and recombine the six herbs in DSS, to explore the further mechanism and to establish the potential active components.

In conclusion, this study only demonstrates that DSS has renoprotection against UUO-induced renal fibrosis, this activity possibly via attenuating tissue hypoxia and promoting autophagy, but the mechanism should be executed for further research.

## Figures and Tables

**Table 1:** Danggui Shaoyao San components.

Chinese term	Generic name	Scientific name	Product lot
Danggui	*Angelica sinensis *(Oliv.) Diels	Angelicae sinensis radix	180413
Baishao	*Paeonia lactiflora *Pall.	Paeoniae radix alba	180323
Chuanxiong	*Ligusticum chuanxiong* Hort.	Chuanxiong rhizoma	180413
Fuling	*Poria cocos* (Schw.) Wolf	Poria	180516
Baizhu	*Atractylodes macrocephala* Koidz.	Atractylodis macrocephalae rhizoma	180418
Zexie	*Alisma orientale* (Sam.) Juzep.	Alismatis rhizoma	180119

## Data Availability

The data used to support the findings of this study are available from the corresponding author upon request.
